# CCL2 is transcriptionally controlled by the lysosomal protease cathepsin S in a CD74-dependent manner

**DOI:** 10.18632/oncotarget.5065

**Published:** 2015-08-22

**Authors:** Richard D.A. Wilkinson, Sinead M. Magorrian, Rich Williams, Andrew Young, Donna M. Small, Christopher J. Scott, Roberta E. Burden

**Affiliations:** ^1^ Molecular Therapeutics, School of Pharmacy, Queen's University Belfast, Belfast, BT9 7BL; ^2^ Centre for Cancer Research and Cell Biology, Queen's University Belfast, Belfast, BT9 7BL

**Keywords:** protease, microenvironment, inflammation, chemokine, cancer

## Abstract

Cathepsins S (CatS) has been implicated in numerous tumourigenic processes and here we document for the first time its involvement in CCL2 regulation within the tumour microenvironment. Analysis of syngeneic tumours highlighted reduced infiltrating macrophages in CatS depleted tumours. Interrogation of tumours and serum revealed genetic ablation of CatS leads to the depletion of several pro-inflammatory chemokines, most notably, CCL2. This observation was validated *in vitro*, where shRNA depletion of CatS resulted in reduced CCL2 expression. This regulation is transcriptionally mediated, as evident from RT-PCR analysis and CCL2 promoter studies. We revealed that CatS regulation of CCL2 is modulated through CD74 (also known as the invariant chain), a known substrate of CatS and a mediator of NFkB activity. Furthermore, CatS and CCL2 show a strong clinical correlation in brain, breast and colon tumours. In summary, these results highlight a novel mechanism by which CatS controls CCL2, which may present a useful pharmacodynamic marker for CatS inhibition.

## INTRODUCTION

Unraveling the complexity of interactions within the microenvironment has become a major focus of biomedical research within recent years. Cellular communications are critical mediators of numerous diseases including fibrosis, myelodysplasia and cancer, where normal cells such as immune cells are recruited and/or reprogrammed to promote disease progression.

CatS is a lysosomal cysteine protease, the expression of which has been associated with a number of inflammatory diseases including rheumatoid arthritis, atherosclerosis and cancer. CatS expression has been shown to be elevated within tissues from these conditions, with genetic ablation or therapeutic targeting strategies diminishing disease severity and/or progression [1–4]. We have previously shown that CatS expression is associated with a number of malignancies including astrocytomas and colorectal carcinomas, and that depletion of CatS can result in the attenuation of tumourigenesis [5–8]. Using therapeutic targeting strategies, we have identified that CatS is a key mediator of tumour invasion and angiogenesis in tumourigenesis [6, 9, 10].

Recent studies have interrogated the impact of CatS with regards to the tumour microenvironment. We have previously examined the contribution of tumour-derived and microenvironment-derived CatS to colorectal tumourigenesis using a stratified depletion approach. We identified that tumour-derived and microenvironment-derived CatS both have significant roles to play in facilitating tumour growth, through proliferation, apoptosis and angiogenesis [8]. Furthermore, the importance of macrophage-derived CatS has also been examined, with studies demonstrating its contribution in murine tumour model growth, and more recently in mediating breast-to-brain metastasis [11, 12].

Extensive work has been carried out to identify protease substrates in order to elucidate protease-specific roles within biological processes. Previous studies have identified multiple CatS substrates such as the angiogenic proteins laminin, arresten, canstatin and endostatin [13, 14]. In this investigation, we examined a possible role of CatS in the regulation of tumourigenic factors. By utilizing commercial antibody arrays, we performed medium throughput, focused screens of tumourigenic proteins in order to identify those exhibiting altered expression due to CatS depletion in murine models and cell lines. We have ascertained that CatS can regulate the expression of several pro-tumourigenic factors, most notably the pro-inflammatory chemokine, CCL2. We showed that this regulation appears to occur through CatS cleavage of CD74, which in turn transcriptionally regulates CCL2 expression, through the activation of NFkB. Interrogation of patient microarray datasets has confirmed the clinical correlation between CatS and CCL2, highlighting physiological significance and suggests that CCL2 may be a potential biomarker of CatS activity within disease.

## RESULTS

### CatS alters stromal cell migration

**Figure 1:**
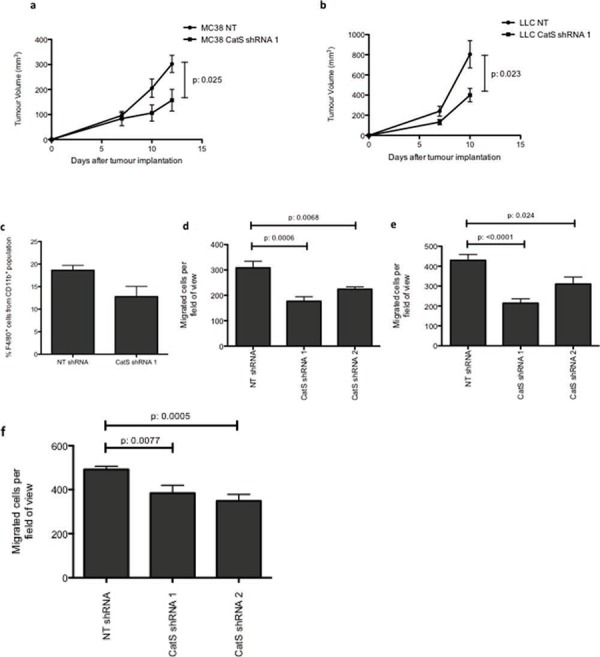
CatS ablation reduces tumour growth and macrophage recruitment MC38 and LLC cells were stably transduced with lentiviral vectors expressing non-targeting (NT) control or CatS targeting shRNA sequences. **a.** MC38 NT control and CatS shRNA expressing cells were implanted into wild-type mice, with tumour growth was monitored and recorded as indicated. **b.** LLC NT control and CatS shRNA expressing cells were implanted into wild-type mice, with tumour growth monitored and recorded as indicated. **c.** LLC tumours were harvested 10 days post-implantation and subjected to flow cytometry to ascertain the number of F4/80^+^ cells. All *in vivo* experiments are indicative of 12 tumours, with data representative of the mean ± SEM. **d.** Raw264.7 cell migration was examined in response to tumour conditioned media from MC38 NT control and CatS shRNA cells (*n* = 3). **e.** Raw264.7 cell migration was also examined in response to tumour conditioned media from LLC NT control and CatS shRNA cells (*n* = 3). **f.** NIH3T3 fibroblast migration was examined in response to conditioned media from MC38 NT control and CatS shRNA expressing cells (*n* = 3). All data is representative of the mean ± SEM.

We have previously shown that tumour-derived CatS is important for the growth of tumours *in vivo* [8]. In this current study we wished to interrogate in more depth the impact of tumour-derived CatS on the tumour microenvironment. MC38 colon carcinoma cells expressing non-targeting control (NT) and CatS shRNA constructs (Supplementary Fig. 1) were grown in wild type C57BL/6 mice and macrophage infiltration was examined by flow cytometry. Whilst tumour cells lacking CatS grew more slowly than control cells expressing the protease, in agreement with our previous findings (Fig. 1a), a difference in infiltrating macrophages was difficult to interrogate by flow cytometry due to the small but highly encapsulated nature of these tumours (data not shown). Therefore, we examined the impact of CatS on an alternative syngeneic model using LLC lung carcinoma tumour cells, generated to express non-targeting control and CatS targeting shRNA constructs (Supplementary Fig. 2). In agreement with our MC38 model, diminished CatS levels in the LLC cells attenuated tumour growth (Fig. 1b). Subsequent analysis by flow cytometry revealed a marked reduction in macrophage infiltration to the tumour (29% reduction) (Fig. 1c).

We then wanted to determine if this effect was preserved *in vitro*, and if this reduced infiltration observed in the complex cellular milieu of the tumour microenvironment was a consequence of specific CatS depletion in the tumour cells. Therefore, conditioned media was harvested from non-targeting control and CatS shRNA expressing cells, and used as a chemoattractant in *in vitro* migration assays using murine monocyte-derived macrophages. Macrophage migration was significantly impaired when conditioned media collected from CatS shRNA expressing cells (in both MC38 and LLC cell line models) was used as a chemoattractant in comparison to controls (Fig. 1d, 1e). We also examined fibroblast migration and found that this too was significantly diminished when CatS shRNA conditioned media was used as a chemoattractant (Fig. 1f).

### CatS controls expression of pro-inflammatory chemokines and fibroblast chemoattractant proteins

**Figure 2:**
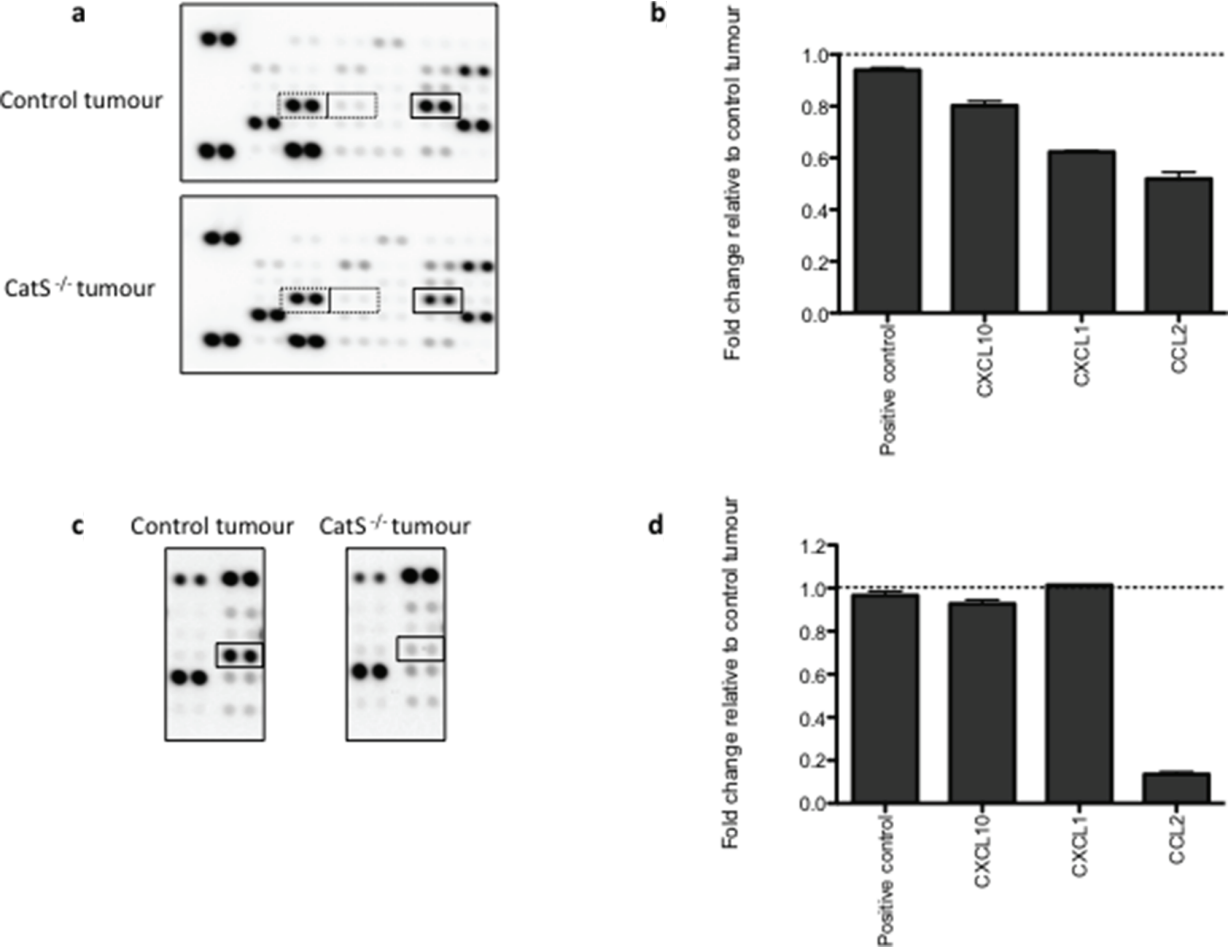
Pro-inflammatory chemokine expression levels are altered in the absence of CatS MC38 NT control cells were implanted in wild-type mice, with CatS shRNA expressing cells implanted into CatS^−/−^ mice in order to identify changes in protein expression due to diminished CatS expression. Tumours and serum were extracted 12 days after implantation. **a.** Interrogation of tumour lysates by antibody array, identified altered expression of CXCL10, CXCL1 and CCL2 (highlighted by the dashed boxes from left to right). **b.** Densitometry analysis of antibody arrays enabled quantification of expression changes of CXCL10, CXCL1 and CCL2. **c.** Serum samples were also interrogated by antibody array to determine changes based on CatS expression status. **d.** Densitometry analysis enabled quantification of the changes in CCL2 expression, made relative to mice bearing the control tumours.

The observation that CatS depletion can attenuate macrophage and fibroblast migration, has revealed a novel and uncharacterized role for CatS in tumourigenesis. We have previously observed the altered expression of angiogenic proteins such as FGF in CatS depleted tumours [8]. In order to elucidate the mechanism by which CatS mediates cellular recruitment, we decided to examine changes in protein expression using commercially available antibody arrays. This allowed us to interrogate if any factors implicated in macrophage recruitment or fibroblast migration were altered as a result of CatS ablation. Antibody array analysis of protein lysates extracted from the MC38 tumours, identified several proteins deregulated as a result of CatS repression. In particular, the absence of CatS correlated with a reduction in several pro-inflammatory chemokines such as CXCL10, CXCL1 and in particular, CCL2 (Fig. 2a, 2b). Interrogation of this data also revealed the deregulation of multiple chemokines that have been postulated to be fibroblast chemoattractants, including TGFβ, previously associated with CatS and fibroblasts in myocardial infarctions [15] (Supplementary Fig. 3). Furthermore, analysis of serum samples from MC38 tumour bearing mice also revealed a notable reduction in CCL2 levels, whereas serum levels of CXCL10 and CXCL1 were unaffected (Fig. 2c, 2d).

### CatS regulates the expression of the pro-inflammatory chemokine CCL2

**Figure 3:**
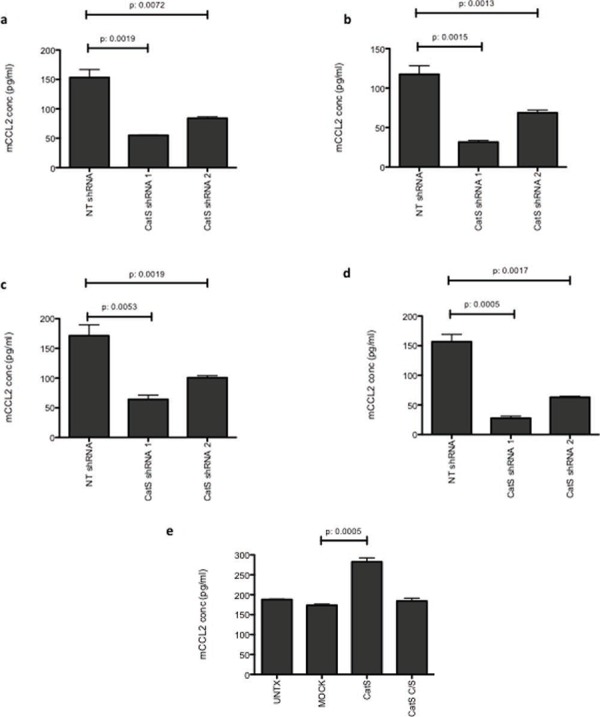
CCL2 expression is correlated with CatS CCL2 expression was measured by ELISA in **a.** MC38 cell lysates, **b.** MC38 supernatants, **c.** LLC cell lysates and **d.** LLC supernatants. **e.** Transient overexpression of CatS in MC38 cells led to the significant induction of CCL2 expression, whereas overexpression of catalytically inactive CatS (CatS C/S) had no impact on CCL2 when measured by ELISA. All experiments are representative of *n* = 3 and data is representative of the mean ± SEM.

In order to validate results obtained in the antibody array, MC38 and LLC CatS shRNA cells were subjected to analysis *in vitro*. Interrogation of both cell lysates and supernatants revealed that CCL2 expression levels were significantly reduced when CatS expression was depleted by shRNA (Fig. 3a–3d). By observing altered expression of both intracellular and extracellular CCL2, this suggested that CatS was not simply altering chemokine cellular secretion mechanisms and that additional mechanisms of control must be involved. To provide further confidence in this effect, we then examined if the overexpression of CatS could induce CCL2 expression levels. MC38 cells were transfected with constructs encoding murine CatS (CatS) or an inactive mutant version where the catalytic cysteine residue was mutated to a serine residue (CatS C/S) (Supplementary Fig. 4). Analysis of CCL2 by ELISA showed that CatS had the ability to induce CCL2 expression and this was dependent upon the catalytic activity of the protease, given that overexpression of CatS C/S had no apparent effect (Fig. 3e). Taken together, this data would suggest that CatS can control CCL2 expression within different tumour cell types and that proteolytic activity of the protease is important in mediating this effect.

### CatS regulation of CCL2 attenuates macrophage migration

**Figure 4:**
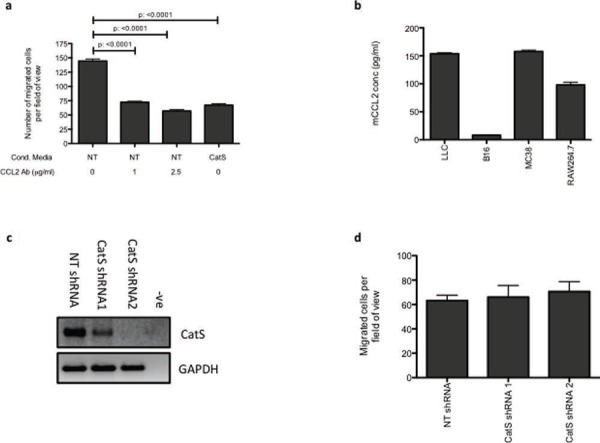
CatS regulation of CCL2 is responsible for altered macrophage recruitment **a.** Raw264.7 cell migration was examined using tumour cell conditioned media from MC38 NT control (NT) and CatS shRNA (CatS) expressing cells. NT control conditioned media was also incubated with an antagonistic CCL2 antibody to verify the impact of CCL2 on cell migration. **b.** The lack of CCL2 expression in B16-F10 cells, was verified by ELISA, in comparison to other cell lines utilized in this work. **c.** B16-F10 cells were engineered to stably express the NT control and CatS shRNA constructs and knockdown of CatS expression was verified by RT-PCR. GAPDH amplification was used as an internal control to ensure equal loading. **d.** Raw264.7 cell migration using conditioned media harvested from B16-F10 NT control and CatS shRNA expressing cells was ascertained. All experiments are representative of *n* = 3 and data is representative of the mean ± SEM.

One of the predominant roles of CCL2 is the recruitment of monocytes/macrophages in a plethora of inflammatory diseases, including cancer [16]. To verify the importance of the CatS-CCL2 axis, macrophage migration assays were also performed in the presence of a CCL2 antagonistic antibody. In MC38 non-targeting control conditioned media, there was a dose-dependent decrease in macrophage migration in the presence of the antagonistic antibody, which was comparable to the impact of conditioned media from MC38 CatS shRNA cells (Fig. 4a).

In order to confirm the impact of CatS on macrophage migration, B16 melanoma cells, which lack CCL2, were utilised and verified by ELISA (Fig. 4b). B16 stable cells lines expressing CatS shRNA constructs were established (Fig. 4c) and migration assays were performed using conditioned media harvested from these cells. Despite CatS levels being substantially reduced by the shRNA sequences, B16 conditioned media had no effect on macrophage migration, irrespective of the CatS status of the cells (Fig. 4d). These findings therefore further confirm the association between CatS and CCL2 is important for macrophage migration.

### CatS transcriptionally regulates CCL2 expression

**Figure 5:**
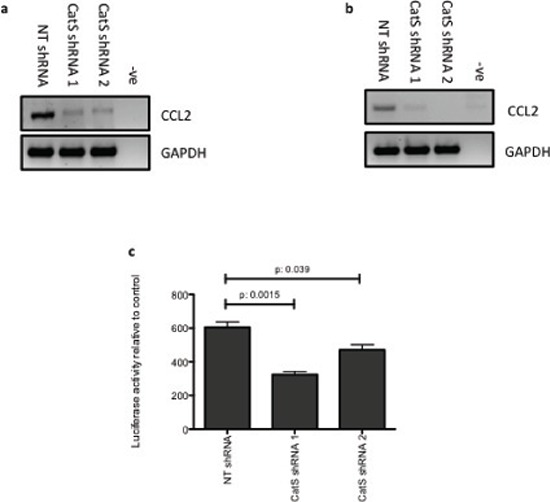
CatS can transcriptionally regulate CCL2 expression RT-PCR analysis of CCL2 expression in **a.** MC38 and **b.** LLC cells expressing CatS shRNA constructs, with GAPDH amplification used as an internal control. **c.** MC38 cells were transiently transfected with a CCL2 promoter construct containing a luciferase reporter element. Luciferase activity was measured 24 hrs following transfection using the Dual-Glo^®^ luciferase assay system. All experiments are representative of *n* = 3 and data is representative of the mean ± SEM.

Having evaluated CCL2 depletion in colon and lung carcinoma cell lines and determined that CCL2 expression levels were regulated both intracellularly and extracellularly, we wanted to examine in more detail how CatS was regulating CCL2. Using MC38 and LLC shRNA cell lines, we assessed expression of CCL2 by RT-PCR and determined that CCL2 was transcriptionally regulated by CatS (Fig. 5a, 5b). Furthermore, these findings were confirmed by transfecting MC38 cells with a luciferase construct encoding the CCL2 promoter region. Luciferase activity was significantly attenuated in cells expressing the CatS shRNA compared to the non-targeting control cells (Fig. 5c). Collectively, these experiments confirmed that CatS has the ability to function as a transcriptional regulator of CCL2 expression.

### CatS regulates CCL2 expression by modulation of CD74 processing

**Figure 6:**
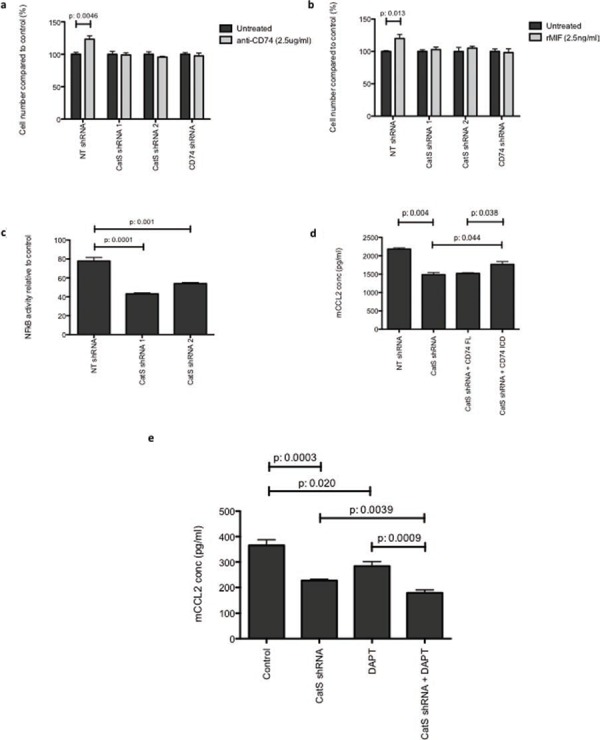
CatS regulation of CCL2 is mediated by CD74 MTT assay was used to measure cell viability, following treatment of MC38 cells with **a.** CD74 agonistic antibody, or **b.** CD74 ligand, MIF. Absorbance was measured at 570 nm and results expressed relative to the untreated control. **c.** Transient transfection of a NFkB luciferase construct in MC38 cells. Luciferase activity was measured 24 hrs following transfection using the Dual-Glo^®^ luciferase assay system. **d.** The impact of transient overexpression of full length CD74 (CD74-FL) and CD74 intracellular domain (CD74-ICD) on CCL2 expression in MC38 cells was determined by ELISA. **e.** MC38 cells were examined for alterations in CCL2 production by ELISA due to expression of the CatS shRNA, following treatment with DAPT, or both in combination. All experiments are representative of n = 3 and data is representative of the mean ± SEM.

Previous studies have implicated the cell surface transmembrane protein CD74 in the regulation of pro-inflammatory chemokines, such as CCL2. Proteolytic cleavage of an intracellular domain fragment (ICD) enables its translocation to the cell nucleus where it promotes NFkB activity [17]. CD74 has been well documented as a chaperone that facilitates stability of the MHC-II heterodimer, which can be cleaved by CatS prior to antigen presentation [18]. To interrogate a link between CatS, CCL2 and CD74, MC38 cells were treated with an agonistic CD74 antibody. Previous studies have identified that the activation of CD74 with this antibody results in the induction of cell proliferation [19]. Our results show that cells expressing CatS display increased proliferation when treated with the agonistic CD74 antibody, whereas there was no effect on cells lacking CatS (Fig. 6a). Likewise, when CD74 expression was depleted by shRNA (Supplementary Fig. 5), the agonistic antibody had no effect (Fig. 6a). In a similar manner, cells treated with the CD74 ligand MIF, also displayed an increase in proliferation compared to cells depleted in either CatS or CD74 (Fig. 6b). Furthermore, given the association between CCL2 expression and NFkB activity, luciferase assays were performed using an NFkB reporter construct transfected into MC38 cell lines. Cells expressing CatS shRNA sequences displayed significantly reduced NFkB activity in comparison to the non-targeting control expressing cells (Fig. 6c).

To further examine the role of CD74 in CatS regulation of CCL2, MC38 cells were transfected with CD74 expression constructs encoding the full-length transmembrane (CD74-FL) and intracellular domain (CD74-ICD, which lacks the transmembrane and endosomal domains) proteins (Supplementary Fig. 6). We hypothesized that overexpression of CD74-FL in CatS depleted cells would have no impact on CCL2 expression level as the protease was not present to facilitate the cleavage and promote release of CD74-ICD. Indeed we found that the reduced CCL2 production caused by depletion in CatS was unable to be rescued by over-expression of the CD74-FL protein. However, the over-expression of the CD74-ICD did induce a significant increase in CCL2 expression, demonstrating that its activity is independent of CatS and in keeping with previous reports that it can constitutively interact with NFkB, requiring no endosomal processing (Fig. 6d).

Previous studies has also implicated the multimeric protein complex, gamma secretase, with the proteolytic release of the CD74-ICD fragment, following CatS cleavage of the endosomal domain of CD74. Here, we have shown that in MC38 colon carcinoma cells, targeting of CatS (using shRNA) or gamma secretase (using DAPT), results in the attenuation of CCL2 protein expression, in agreement with previous observations [20, 21]. However, our results have also shown that the greatest impact on CCL2 expression appears to be mediated via depletion of CatS, with an enhanced effect observed when combined with gamma secretase inhibition using DAPT (Fig. 6e). Collectively, these findings would suggest that the gamma secretase cleavage does occur downstream of the CatS cleavage event.

### CCL2 expression is a potential biomarker for CatS activity

**Figure 7:**
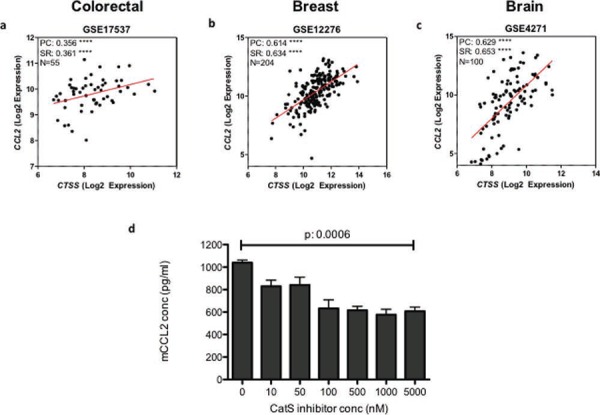
CatS and CCL2 expression are correlated in human tumour samples Gene array analysis examining the correlation between CatS and CCL2 in human tumour datasets representing **a.** colorectal, **b.** breast and **c.** brain tumours. **d.** MC38 cells were treated with an increasing concentration of CatS inhibitor 6 and the impact on CCL2 expression in the supernatants was measured by ELISA. Experiments are representative of *n* = 3 and data is representative of the mean ± SEM. PC: Pearson coefficient, SR: Spearman rank.

Finally, we wanted to investigate the significance of these findings through the examination of clinical material. A number of publically available gene array datasets were studied in order to delineate the clinical relationship between CatS and CCL2. Analysis was performed using a range of human tumour types (colorectal, breast and brain carcinomas) and a significant correlation between CatS and CCL2 expression was observed in all tumour datasets examined (Fig. 7a–7c). In our previous work, we have documented an association between CatS expression and patient survival in both brain and colorectal carcinomas, both of which identified CatS as a poor prognostic marker [7, 22]. Studies have also proposed CCL2 as a poor prognostic marker in multiple different tumour types, in particular breast cancer [23, 24]. Using CatS selective small molecule inhibitor compound 6 (Merck-Frosst) [25] (Supplementary Fig. 7), we have demonstrated a significant dose-dependent reduction in CCL2 expression following treatment with the inhibitor, adding further support to our hypothesis that CCL2 is a potential biomarker of CatS activity (Fig. 7d).

## DISCUSSION

CatS has been implicated in the regulation of tumourigenesis by facilitating invasion and angiogenesis [5, 6, 13]. Numerous studies have also shown that CatS has a significant correlation with other inflammatory diseases. Given the inherently inflammatory nature of tumours, we wanted to investigate the mechanisms by which this protease contributes to tumourigenesis. This work details for the first time the potential for CatS to function as a transcriptional mediator of gene expression. Whilst previous publications have documented roles for CatS in tumourigenesis, there has been little evidence to support specific mechanisms by which the protease mediates the disease. The main findings from our studies are that tumour-derived CatS can transcriptionally regulate the pro-inflammatory chemokine CCL2, in a CD74-dependent manner and influence the tumour microenvironment, by altering cellular recruitment of macrophages. As such, this work opens up a plethora of potential novel roles for not only this protease, but other cathepsin family members also, in the mechanistic regulation of disease.

The role of CCL2 as a potent regulator of monocyte/macrophage recruitment has been well documented [26–28]. Eloquent work by Qian *et al*, identified CCL2 as a major mediator of inflammatory monocyte recruitment in breast cancer metastasis and how a specific subtype of macrophages, termed metastasis associated macrophages (MAMs) are preferentially recruited to pulmonary metastasis [24]. Further studies have also implicated CCL2 with roles in multiple inflammatory diseases such as Alzheimer's Disease [29], Multiple Sclerosis [30] and pulmonary fibrosis [31]. Given the association of both CatS and CCL2 in a plethora of inflammatory diseases, we believe that CCL2 may have potential to act as a biomarker for CatS activity in clinical material, given the close correlation in their expression in our clinical sample analysis and preliminary experiments with CatS targeting compounds. The development of therapeutic targeting strategies towards CatS is of particular interest to numerous drug companies and several clinical trials are currently underway evaluating the efficacy of such compounds in diseases such as rheumatoid arthritis, neuropathic pain and psoriasis [32].

This work has delineated that CatS-mediated transcriptional regulation of CCL2, is facilitated, at least in part, through known CatS substrate, CD74. CD74 is a type 1 transmembrane glycoprotein, primarily known for its role as the invariant chain in MHC-II antigen presentation. CatS has been previously implicated with CD74 processing through the mediation of MHC-II antigen presentation, where CD74 cleavage is essential for maintaining stability of the MHC-II heterodimer and facilitating peptide loading [18, 33]. CD74 and its ligand, MIF (macrophage migration inhibitory factor) have previously been implicated in inflammatory diseases [34].

**Figure 8:**
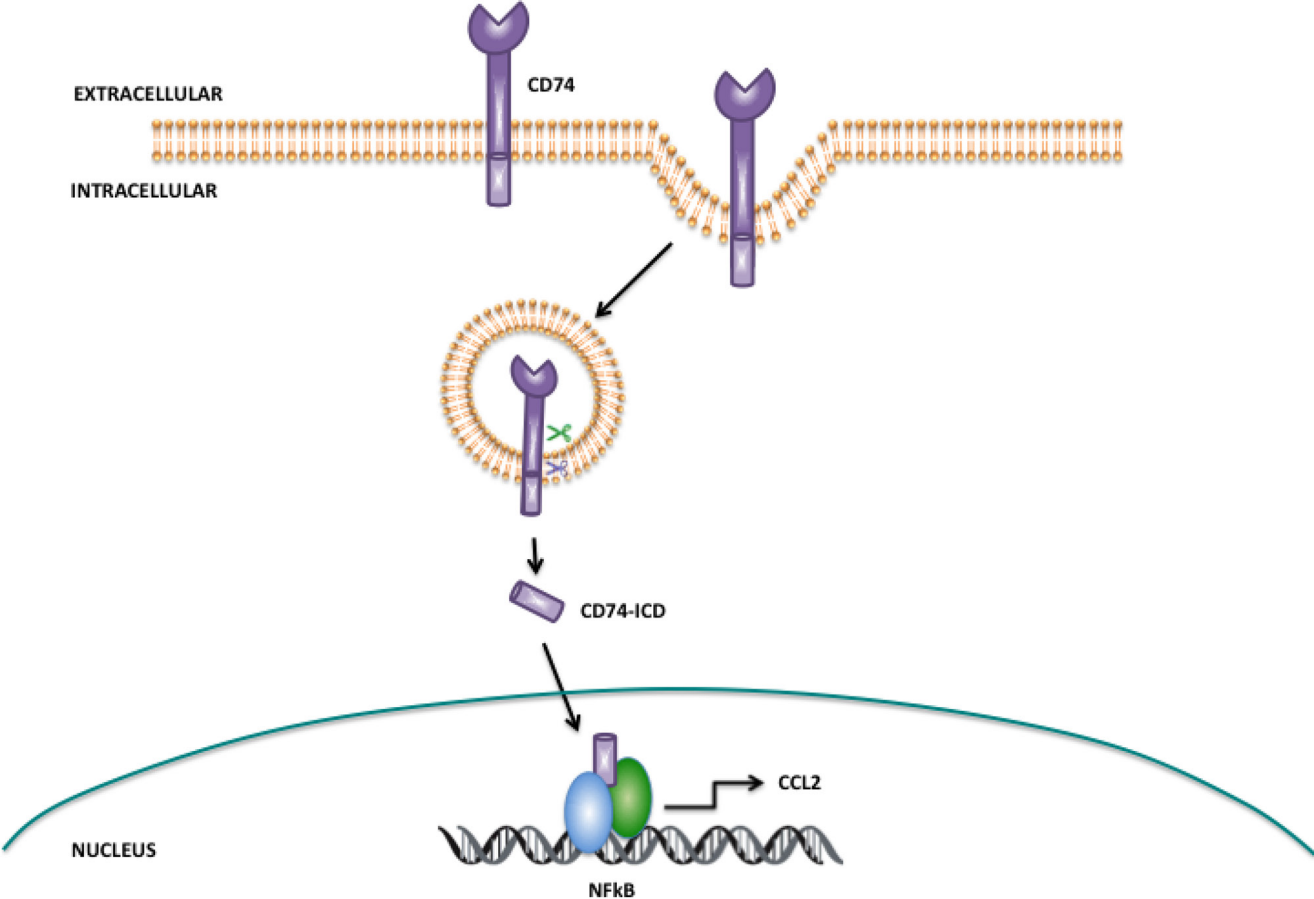
Proposed mechanism of action for CatS mediated regulation of CCL2 via CD74 CD74 is internalized within endocytic vesicles, where the extracellular domain resides within the vesicle and the intracellular domain (CD74-ICD) extends into the cytoplasm. CatS within the endocytic vesicle has been proposed as a key protease responsible for the cleavage of the CD74 within the endosome (green scissors), whereas gamma secretase has been implicated in regulated intramembrane proteolysis of the transmembrane domain of CD74 resulting in liberation of CD74-ICD (blue scissors). Released CD74-ICD can translocate and interact with NFkB in the nucleus where it regulates the expression of chemokines such as CCL2. Adapted from Martín-Ventura, J.L. *et al* [20].

Studies by the Shachar group have extensively examined CD74 and have identified that it is a member of the regulated intramembrane proteolysis (RIP) protein family, with sequential proteolytic cleavage of CD74 resulting in the liberation of the CD74 intracellular domain (CD74-ICD), which can initiate NFkB activation and CCL2 expression (as illustrated in Fig. 8). Further research suggested that CatS mediated cleavage of CD74 is required prior to the intramembrane release of the ICD by gamma secretase; inhibition of CatS with a small-molecule inhibitor results in the accumulation of CD74 intermediate fragments and prevents the release of CD74-ICD. Furthermore, deletion of the luminal domain of CD74 (which contains the CatS cleavage site), enables more efficient release of the ICD fragment. Collectively this indicates that CatS must cleave the luminal domain of CD74 first in order for release of the ICD to be mediated by gamma secretase [22]. Our results presented here are consistent with this previous research, but clearly other mechanisms for modulation of CCL2 are likely to exist.

Whilst previous studies have associated CatS expression with tumour infiltrating macrophages [8, 11, 12, 35], there has been limited work to identify the mechanistic involvement of this protease in mediating tumourigenesis. Recent work by Shi *et al*, has identified a reduction in macrophage recruitment using CatS null mice in neointimal hyperplasia studies [36]. This work is in agreement with our observations that CatS can regulate macrophage recruitment towards inflammatory areas in disease, but we believe that we have added to this recent work through the identification of CCL2 as a key chemokine involved in the process, determining that CatS mediates this effect through the transcriptional regulation of CCL2.

With regards to our novel observations on the impact of CatS on fibroblast migration, the mechanistic regulation of this observation is largely unknown. Historically, there has been little evidence to associate CatS with fibroblasts, but recent studies have suggested that CatS plays an important role in mediating fibroblast transdifferentiation in myocardial infarctions [15]. Our observations that CatS deregulates several reported fibroblast chemokines warrants further investigation in order to dissect the mechanism behind these findings.

Collectively, the work outlined in this study provides novel evidence for mechanistic roles of CatS in diseases such as cancer. We hypothesize that other inflammatory based disorders such as cardiovascular disease, may also exhibit similar mechanisms in relation to CatS function. Previous work has suggested that proteases such as CatS can mediate processing of chemokine function in order to regulate their activity [37], but this work shows for the first time that CatS can function as a transcriptional regulator of protein expression. CCL2 could be a potential biomarker for CatS activity in disease, it is likely that other factors may also exist but these would require further in depth analysis. Together, these findings reinforce the rational for understanding the mechanistic role of these proteases in disease when considering them as therapeutic targets.

## MATERIALS AND METHODS

### Cell culture experiments

293T human embryonic kidney cells (ATCC), NIH-3T3 fibroblasts (ATCC), MC38 colon adenocarcinoma cells (S Rosenberg, NCI), B16-F10 murine melanoma cells (ATCC) and RAW 264.7 leukaemic monocyte macrophage cells (ATCC) were cultured using DMEM supplemented with 10% FBS (Life Technologies, UK), 100 μg/ml penicillin and 100 μg/ml streptomycin (Life Technologies, UK). The Lewis lung carcinoma line (LLC) (ATCC) was cultured using RPMI 1640 (Life Technologies, UK) supplemented with 10% FBS (Life Technologies, UK), 100 μg/ml penicillin and 100 μg/ml streptomycin (Life Technologies, UK). All cells were maintained in a humidified environment containing 5% CO_2_ at 37°C. Experiments within which cells were treated with the gamma secretase inhibitor DAPT (Tocris), cells were seeded in 96 well plates and treated with 50 μM DAPT for 5 hours, prior to collection of cell supernatants for downstream analysis.

### Lentiviral generation of stable shRNA lines

CatS targeting shRNA constructs and non-targeting controls within the pLKO.1 plasmid were purchased, alongside the packaging plasmid mix for lentiviral transduction (Sigma Aldrich, UK). Viral particles were produced by transfection of the constructs into the 293T packaging cell line using GeneJuice (Merck Millipore, UK). After 48 hrs, viral supernatants were collected, filtered through 0.45 μm filters and supplemented with 6 μg/ml PolyBrene (Sigma Aldrich, UK) before being applied to the cells of interest for 4 hrs. Following this, the lentiviral particle solution was removed from the cells and replaced with fresh full media for 24 hrs before selection of a stable cell line in puromycin. Successful diminution of CatS expression was verified by RT-PCR.

### Reverse transcriptase-PCR

RNA was extracted from cell lines using STAT60 (Biogenesis, UK) according to manufacturers instructions. mRNA was converted to cDNA using the ImProm-II Reverse Transcriptase system (Promega, UK) and target sequences were amplified by conventional PCR using RedTaq (Sigma Aldrich, UK) with gene specific primers. All PCR products were visualized using agarose gel electrophoresis on a 1% gel.

### *In vivo* experiments

All mice used in these experiments were aged between 8 and 12 weeks with housing and experimentation carried out in accordance with the Animal (Scientific Procedures) Act 1986, following current UKCCCR guidelines and approved by the Ethical Review Committee within Queen's University Belfast. C57BL/6 mice were purchased from Charles Rivers Laboratories and CatS^−/−^ mice were obtained from J. A. Joyce, Memorial Sloan Kettering Cancer Center, New York with permission from H Chapman, UCSF. Mice were subcutaneously injected with 5 × 10^5^ MC38 or LLC cells mixed with reduced growth factor Matrigel (final concentration: 4 mg/ml) (BD Biosciences, UK). Tumor volumes were calculated with digital calipers, using the formula, V = a × b2 × π/6, with a and b representing the longer and shorter diameter of the tumor, respectively. Data was presented as mean tumor burden (mm^3^) per group ± SEM.

### Flow cytometry

Flow cytometry experiments were performed as previously described [8]. Briefly, tumors were disaggregated and digested in 0.125% trypsin for 20 min at 37°C. The resultant cell suspension was filtered through a nylon mesh (BD Biosciences, UK) to remove debris, washed, centrifuged and resuspended in sterile PBS. Cells were blocked on ice for 10 min using Mouse BD Fc block (BD Biosciences, UK) and then stained using anti-mouse F4/80 conjugated to PE-Cy7 (EBioscience, UK) and anti-mouse CD11b conjugated to APC (BD Biosciences, UK). Flow cytometry was performed with a BD FacsAria II with FacsDiva software and data analysis was performed using Flowjo.

### Migration assays

Raw264.7 and NIH3T3 cells were analysed for their ability to migrate through 8.0 μm transwell inserts (Corning, USA). Cells were resuspended to 5 × 10^5^/ml in serum free media with 200 μl added to the inner chamber of each transwell insert. Conditioned media from the appropriate cell line (700 μl) was added to the lower chamber to act as a chemoattractant and cells were incubated at 37°C with 5% CO_2_ for 16 hrs. Non-migrated cells were removed and migrated cells were fixed in Carnoy's fixative for 15 mins. After drying, the nuclei of the migrated cells were stained with Hoechst 33258 (50 ng/mL) in PBS for 30 mins at room temperature. The chamber insert was washed twice in PBS and mounted in Citifluor, and migrated cells were viewed and counted with a Nikon Eclipse TE300 fluorescent microscope. All assays were done in triplicate and 10 digital images of representative fields from each of the triplicate membranes were taken using a Nikon DXM1200 digital camera at magnification of x20. Results were analyzed using Lucia GF 4.60 software by Laboratory Imaging and expressed as the average number of migrated cells per field of view (±SEM).

### Antibody array

Tumour lysates were prepared using RIPA buffer (Sigma-Aldrich, UK) and serum samples were extracted from MC38 tumour bearing mice for analysis using Proteome Profiler Antibody arrays (R&D Systems, UK). Arrays were performed according to manufactures instructions and were visualized and quantified by chemiluminescence using the ChemiDoc XRS imaging system (Bio-Rad Laboratories).

### ELISA

Murine CCL2 ELISAs (R&D Systems, UK) were performed on tumour lysates and supernatants according to manufactures instructions. Briefly, maxisorb 96 well plates were coated with anti-CCL2 capture antibody overnight at room temperature. Plates were washed in PBS-T and blocked in 1% BSA in PBS for 1 hr prior to incubation with the protein sample at room temperature for 2 hrs. Following washing, plates were incubated with the murine CCL2 detection antibody, washed and then incubated with streptavidin-HRP. Following the final wash steps, wells were incubated with the chromogenic substrate tetramethylbenzidine (Calbiochem) for 10 min at room temperature. The reaction was stopped by the addition of 500 mM HCl and absorbance was read at 450 nm.

### Transfections

All transfections were performed using Genejuice (Merck Millipore, UK) according to manufactures instructions. Briefly, cells were seeded and transfected with murine CatS, CatS C/S, CD74-FL or CD74-ICD expression constructs. 48 hrs following transfection, cells were harvested for subsequent analysis. CatS constructs had been cloned into pcDNA3.1 incorporating a C-terminal Flag tag, whilst CD74 constructs were a kind gift from Professor Idit Shachar (Weizman Institute, Israel) and were also sub-cloned into pcDNA3.1 to incorporate a C-terminal Flag tag. The catalytic cysteine residue of pcDNA3.1 CatS was mutated to a serine reside using site directed mutagenesis (Agilent Technologies, USA) to give rise to the CatS C/S construct.

### Luciferase assays

Cells were seeded in 6-well plates and transfected with a pGL3 NFkB reporter, Renilla, CCL2 promoter or control plasmid using Genejuice (Merck Millipore, UK) according to manufactures instructions. Cells were incubated for 24 hrs following transfection before lysis with passive lysis buffer and luciferase activity was determined using Dual-Glo^®^ luciferase assay system (Promega, UK) as per manufactures instructions. All experiments were repeated in triplicate a minimum of three times. pGL3 based constructs were a kind gift from Dr Niamh Buckley (Centre for Cancer Research and Cell Biology, Queen's University Belfast) and the CCL2 luciferase promoter construct was purchased from Addgene (Addgene, USA).

### MTT assay

Cell proliferation following treatment with anti-CD74 agonistic antibody (Santa Cruz, USA) or mMIF recombinant protein (R&D Systems, UK) was assessed by MTT assay. Cells were seeded at 1 × 10^4^ per well of a 96-well plate in 200 μl of cell growth medium. Cells were treated with the agonistic antibody (2.5 μg/ml) or MIF protein (2.5 ng/ml) and incubated at 37°C with 5% CO_2_ for 24 hrs. Cells were incubated with 20 μl of 10 mg/ml MTT and returned to the incubator for a further 3 hrs. The media and MTT mixture was carefully removed and the insoluble formazan crystals were dissolved in 50 μl/well of DMSO. Absorbance was measured at 570 nm and the results were expressed as the percentage cell number relative to the untreated control. All tests were done in quintuplicate.

### Co-expression analysis of CCL2 and CTSS

Publically available microarray datasets were obtained from gene expression omnibus (GEO) containing clinical expression profiles in colorectal (GSE17537), breast (GSE12276) and brain (GSE4271) cancers. Data was transformed into Log2 expression and individual probe sets corresponding to desired gene sets identified. For each gene of interest, a median value of expression intensity was obtained from each gene probe set. Gene co-expression was analysed using ‘R’ statistical software program and correlation was evaluated by Pearson coefficient (PC) and Spearman Rank (SR).

### Statistical analysis

Within these experiments, results have been plotted using Prism software (GraphPad, USA) and errors bars have been plotted as the standard error of the mean (SEM). The Student's *t* test has been used to determine the statistical significance between the different samples.

## SUPPLEMENTARY MATERIAL FIGURES


