# Supplementary material for: CCL2 is transcriptionally controlled by the lysosomal protease cathepsin S in a CD74-dependent manner
Source: Oncotarget. 2015 Aug 22;6(30):29725–39. doi: 10.18632/oncotarget.5065 (PMC4745758; doi:10.18632/oncotarget.5065)
Supplement: Supplementary file 1 [file oncotarget-06-29725-s001.pdf]

SUPPLEMENTARY FIGURES

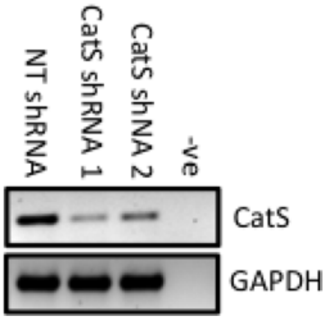

Supplementary Figure S1: Validation of MC38 CatS shRNA cell lines. CatS expression was depleted in MC38 cells using shRNA constructs. The expression of CatS in NT shRNA control cells and the efficacy of CatS shRNA was determined using RT-PCR analysis, with GAPDH amplified as an internal loading control.

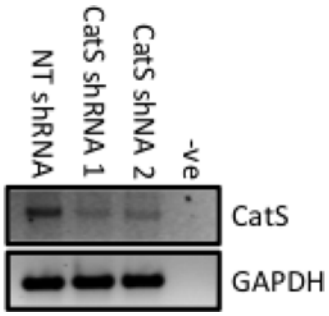

Supplementary Figure S2: Validation of LLC CatS shRNA cell lines. CatS expression was depleted in LLC cells using shRNA constructs. The expression of CatS in NT shRNA control cells and the efficacy of CatS shRNA was determined using RT-PCR analysis, with GAPDH amplified as an internal loading control.

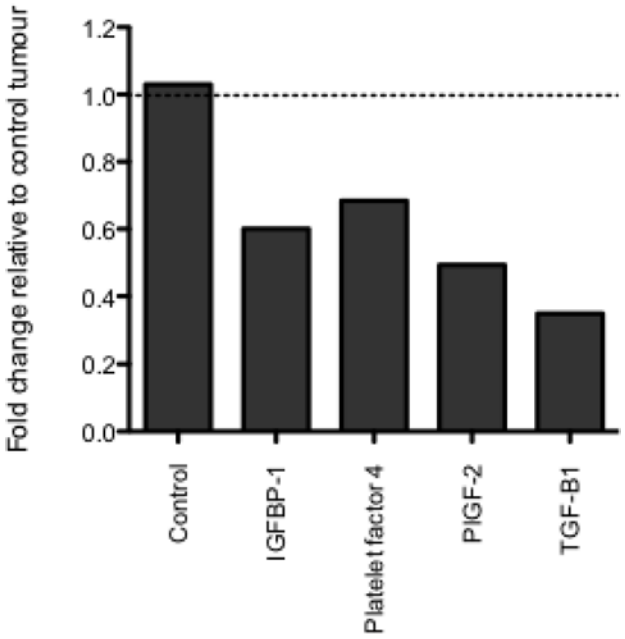

Supplementary Figure S3: Fibroblast chemoattractant proteins deregulated by CatS ablation. Using commercial antibody arrays and MC38 tumour lysates, several proteins reported as fibroblast chemoattractant proteins were identified as being downregulated following CatS ablation. Control tumours were NT control MC38 cells implanted in wild-type mice whereas CatS$^{-/-}$ tumours were CatS shRNA MC38 cells implanted in CatS$^{-/-}$ mice.

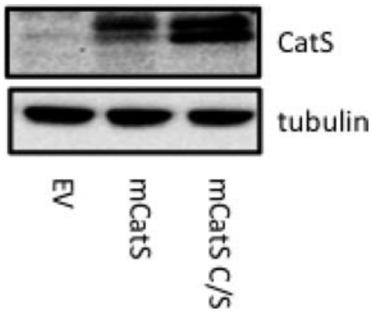

Supplementary Figure S4: Validation of CatS overexpressing cell lines. Murine CatS and CatS C/S constructs incorporating a C-terminal Flag tag were transiently expressed in MC38 cells using Genejuice. 48 hrs post-transfection, cells were harvested and lysates examined by western blotting using an anti-Flag M2 antibody. Tubulin expression was visualized as an internal loading control.

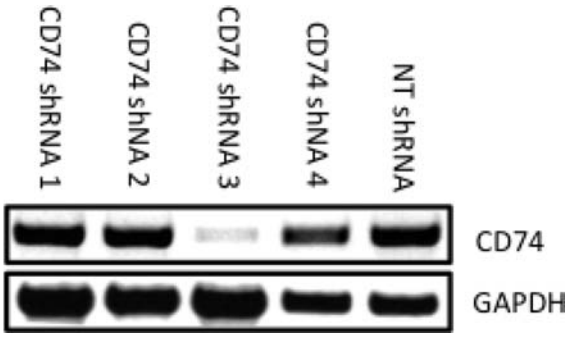

Supplementary Figure S5: Validation of MC38 CD74 shRNA cell lines. CD74 expression was depleted in MC38 cells using shRNA constructs. The expression of CD74 in NT shRNA control cells and the efficacy of CD74 shRNA was determined using RT-PCR analysis, with GAPDH amplified as an internal loading control. CD74 shRNA 3 was used for all subsequent experiments.

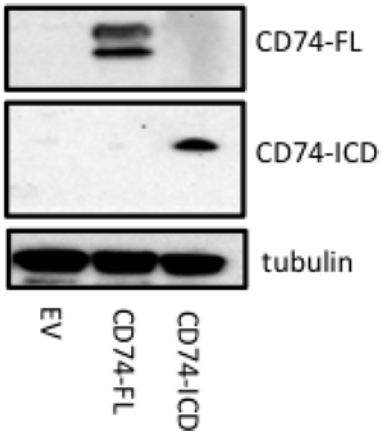

Supplementary Figure S6: Validation of CD74 overexpressing cell lines. Murine CD74-FL (full-length) and CD74-ICD (intracellular domain) constructs incorporating a C-terminal Flag tag were transiently expressed in MC38 cells using Genejuice. 48 hrs post-transfection, cells were harvested and lysates examined by western blotting using an anti-Flag M2 antibody. Tubulin expression was visualized as an internal loading control.

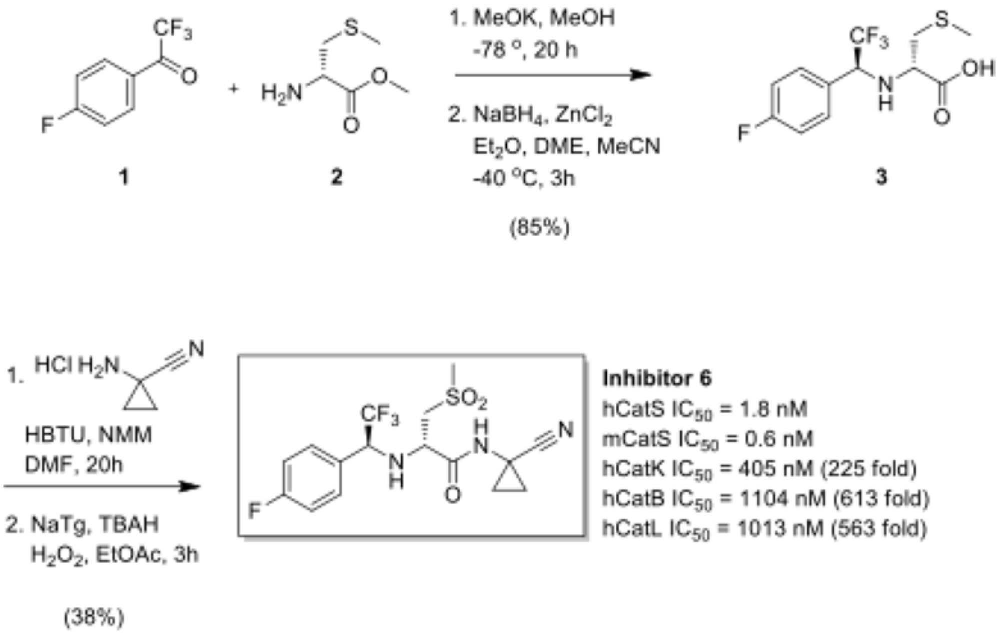

Supplementary Figure S7: Chemical synthesis of CatS inhibitor 6. The synthetic route for Inhibitor 6 (Merck-Frosst) with potency values against mouse and human Cathepsin S plus human Cathepsins K, B and L.
